# Advanced Machine Learning for Comparative Synovial Fluid Analysis in Osteoarthritis and Rheumatoid Arthritis

**DOI:** 10.3390/metabo15020112

**Published:** 2025-02-10

**Authors:** Karolina Krystyna Kopeć, Gabrieleanselmo Uccheddu, Paweł Chodnicki, Antonio Noto, Cristina Piras, Martina Spada, Luigi Atzori, Vassilios Fanos

**Affiliations:** 1 Department of Mechanical, Chemical and Materials Engineering, University of Cagliari, 09123 Cagliari, Italy; 2 School of Residency of Orthopedics and Traumatology, University of Sassari, 07100 Sassari, Italy; g.uccheddu3@studenti.uniss.it; 3 Department of Physical Chemistry, Gdansk University of Technology, 80-233 Gdańsk, Poland; pawel.chodnicki@pg.edu.pl; 4 Department of Biomedical Sciences, University of Cagliari, 09042 Cagliari, Italy; antonio.noto@unica.it (A.N.); cristina.piras@unica.it (C.P.); martina.spada@unica.it (M.S.); luigi.atzori@unica.it (L.A.); 5 Department of Surgical Sciences, University of Cagliari, 09042 Cagliari, Italy; vafanos@tiscali.it

**Keywords:** osteoarthritis, rheumatoid arthritis, machine learning, genetic algorithm

## Abstract

Osteoarthritis (OA) and rheumatoid arthritis (RA) are joint diseases that share similar clinical features but have different etiologies, making a differential diagnosis particularly challenging. **Background/Objectives**: Utilizing advanced machine learning (ML) techniques on metabolomic data, this study aimed to identify key metabolites in synovial fluid (SF) that could aid in distinguishing between OA and RA. **Methods**: Metabolite data from the MetaboLights database (MTBLS564), analyzed using nuclear magnetic resonance (NMR), were processed using normalization, a principal component analysis (PCA), and a partial least squares discriminant analysis (PLS-DA) to reveal prominent clustering. **Results**: Decision forests and random forest classifiers, optimized using genetic algorithms (GAs), highlighted a selection of a few metabolites—primarily glutamine, pyruvate, and proline—with significant discriminative power. A Shapley additive explanations (SHAP) analysis confirmed these metabolites to be pivotal predictors, offering a streamlined approach for clinical diagnostics. **Conclusions**: Our findings suggest that a minimal set of key metabolites can effectively be relied upon to distinguish between OA and RA, supported by an optimized ML model achieving high accuracy. This workflow could streamline diagnostic efficiency and enhance clinical decision-making in rheumatology.

## 1. Introduction

Osteoarthritis (OA) is a degenerative disease that affects every component of joints beginning with cartilage, subchondral bone, synovium, ligaments, and, finally, the outer layers of the capsule [1,2,3,4]. Key risk factors for OA include age, sex, previous joint injuries, obesity, genetic factors, and mechanical issues such as joint malalignment, overuse, and abnormal joint shapes [5,6,7]. Primary OA is characterized by a partially understood pathogenesis. A low degree of joint inflammation is always present, sometimes because of metabolic syndrome [6,8,9]; innate and adaptive immunity also have a role in joint degeneration by further activating the inflammatory cascade [10]. The inflammatory process also has a neurological aspect while the process of joint remodeling goes through neoangiogenesis [6]. 

Rheumatoid arthritis (RA) is an autoimmune disease characterized by joint infiltration of autoimmune antibodies such as the rheumatoid factor (RF) and anti-citrullinated protein antibodies (ACPAs) and inflammatory molecules that mainly target the synovium, cartilage, and subchondral bone, leading to joint pain, swelling, and permanent remodeling, often causing permanent disability [11,12,13]. Risk factors include several gene variants, such as HLA-DRB1, HLA-DR1, and HLA-DR4 [14,15]. Unlike OA, RA can simultaneously target more than one joint, often symmetrically, and its onset is insidious, targeting the small joints [16].

With recent advances in metabolite analysis, such as the pairing of machine learning (ML)-based algorithms with nuclear magnetic resonance (NMR) or liquid chromatography coupled with mass spectrometry (LC-MS), significant advancements have been made in understanding OA and RA. Several studies have leveraged these advanced analytical techniques to discern stage-related and discriminant metabolites in these conditions, other than identifying metabolites that correlate with total joint arthroplasty non-responders, which suggests that metabolic alterations play a huge role before and after the surgical treatment of such pathologies [17,18,19,20]. Bocsa’s research, utilizing LC-MS enhanced by machine learning (ML) algorithms, identified stage-related metabolites in OA (sphingomyelin and phosphatidylcholine), improving our understanding of its progression [18]. A recent article combined the use of NMR with ML-based algorithms such as random forest (RF) and Boruta feature selection (BFS) to identify three principal metabolites (interleukin-6, acetoacetate, and tyrosine) that were suggestive of inflammation in samples of synovial tissue (ST) taken from patients affected by knee OA [21]. Anderson’s work, utilizing NMR, emphasized how specific metabolites such as citrate, glucose, glutamine, and taurine significantly differ between OA and RA synovial fluid, and associated these variations with specific metabolic pathways such as glycolysis and the tricarboxylic acid cycle [17].

In this work, we explore the potential of combining statistical methods with machine learning approaches for the identification of osteoarthritis and rheumatoid arthritis. We utilize a genetic algorithm (GA) as a fine-tuning tool for our model, building upon the dataset previously analyzed by Anderson et al. [17]. This methodology demonstrates how such algorithms can streamline the analysis process by efficiently narrowing down the expansive parameter space. We argue that this approach not only enhances model performance but also has the potential to significantly reduce the time required for similar analyses in other disease contexts. By revisiting this dataset, we aim to offer a new perspective that highlights the value of advanced computational techniques, uncovering more precise and actionable insights into the metabolic foundation of these diseases.

## 2. Materials and Methods

### 2.1. Data Source

The dataset utilized in this study was acquired from the MetaboLights database, specifically, the metabolite profiling dataset MTBLS564. This dataset, accessible at https://www.ebi.ac.uk/metabolights/MTBLS564 (accessed on 20 October 2024), contains metabolomic data derived from synovial fluid samples of patients diagnosed with OA and RA that were acquired using NMR spectroscopy. Synovial fluid (SF) was collected from the knee joints of 10 patients with OA and 14 patients with RA.

### 2.2. Data Preprocessing and Exploratory Analysis

The raw dataset was assessed for missing values and improperly characterized columns, which were removed if they lacked sufficient information. To prepare the data for analysis, five normalization techniques were tested, including Z-score normalization, min–max scaling, max absolute scaling, robust scaling, and combined median + Pareto scaling. The normalization method that provided the best balance of variance and clustering separation was selected for further processing. This step ensured that the data were appropriately scaled for the subsequent analysis steps.

An initial principal component analysis (PCA) was performed to visually explore the potential for separability between the OA and RA groups in a reduced-dimensional space. Although the PCA was not included in the final classification models, it served as a valuable exploratory tool, providing a visual assessment of the dataset’s structure and helping to determine whether clear linear separability existed between the two classes. Following the PCA, a partial least squares discriminant analysis (PLS-DA) was conducted to preliminarily assess the influence of individual metabolite bins on class distinction. The PLS-DA enabled an initial selection of metabolites with significant contributions to group separation, providing insights into features for further analysis.

### 2.3. Machine Learning Workflow

**Figure 1:**
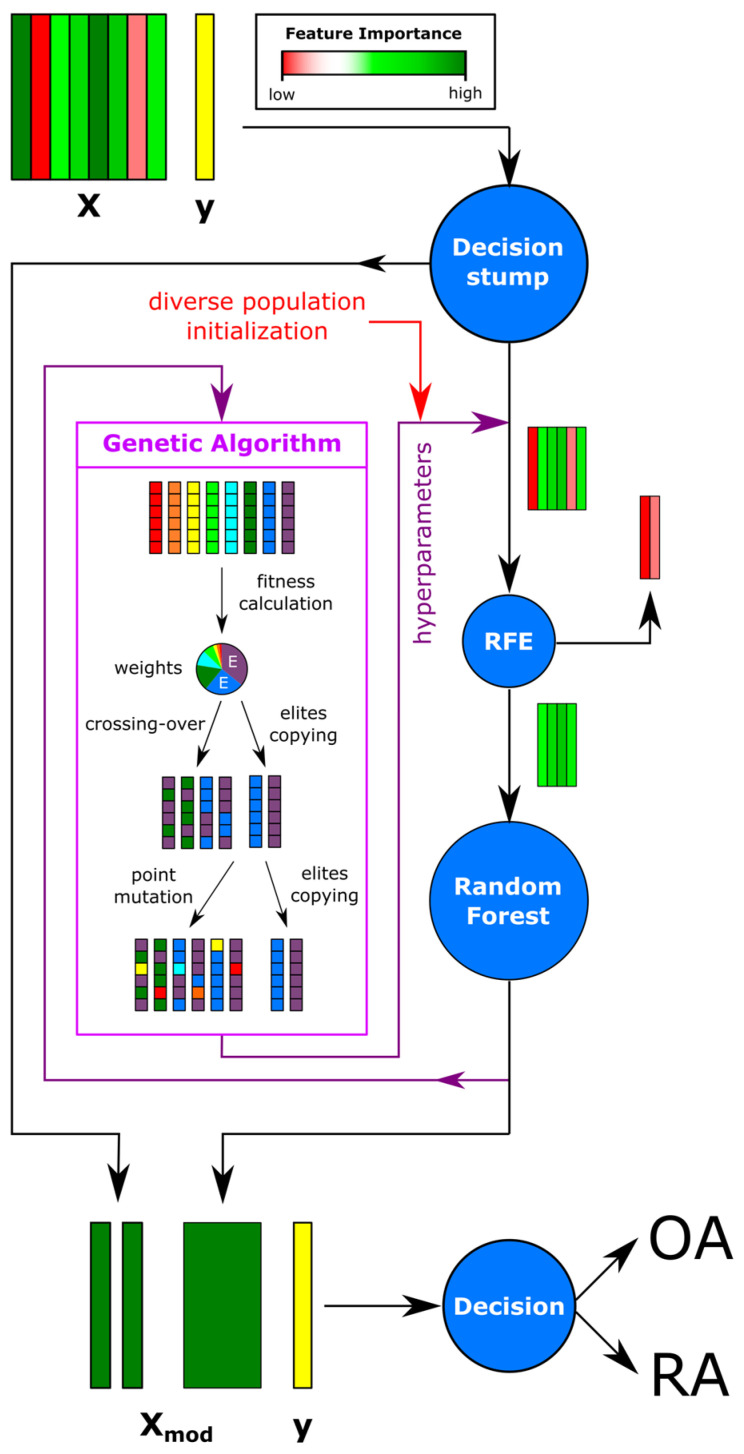
A schematic overview of the study’s diagnostic workflow, integrating a decision stump, reverse feature extraction via support vector machines (SVMs), and a random forest to identify critical metabolite bins distinguishing the disease. The workflow also incorporates a genetic algorithm to optimize the selection of model hyperparameters.

The primary classification and metabolite bin selection workflow is outlined in Figure 1. This workflow integrated multiple machine learning components, including (1) decision stump, (2) recursive feature elimination (RFE), (3) random forest, and (4) genetic algorithm (GA) hyperparameter tuning. Each stage of the workflow was described as follows:

1. **Decision Stump:** A single-level decision tree was employed to individually assess each metabolite bin for its discriminatory power. This approach generated a preliminary ranking of metabolites based on their individual classification performance, serving as the foundation for the subsequent feature selection steps.
2. **RFE:** This is a feature selection technique commonly used in machine learning to enhance model performance by systematically identifying and retaining the most relevant features. It works by recursively training a model, ranking features based on their contribution to the predictive task and eliminating those with the least importance. This iterative process refines the feature set to optimize both model accuracy and complexity.
  In this study, RFE was applied during the later stages of the analysis to remove less relevant metabolite bins, effectively reducing noise in the dataset and improving the robustness of the classification model. The support vector machine (SVM)-based implementation of RFE established a linear decision boundary between classes and determined metabolite bin importance at each step. This refinement process ensured that only top N_RFE_ high-importance metabolites were retained, where N_RFE_ denotes the number of selected features. This enhanced the model’s overall effectiveness and highlighted the necessity of noise reduction to achieve the best performance.
3. **Random Forest Classification:** Using the reduced metabolite bin set, a random forest classifier was trained to differentiate between OA and RA samples. Given the dataset’s limited size, comprising samples from only 24 patients, leave-one-out cross-validation (LOOCV) was employed to evaluate the model’s performance. This approach ensured a robust performance assessment by iteratively using one sample as the test set while training the model using the remaining samples.
  The performance of the random forest classifier was quantified using the Matthews correlation coefficient (MCC), a metric well-suited to evaluating binary classification tasks, particularly in imbalanced datasets. In addition, other performance metrics, including accuracy, precision, recall, F1-score, and ROC-AUC, were also calculated.
  Hyperparameter tuning of the random forest model was conducted to optimize performance, focusing on the following key parameters:
  - **Number of trees (N_est_)**: the total number of decision trees in the forest;
  - **Maximum tree depth (D)**: the maximum depth allowed for each tree;
  - **Minimum samples required to split a node (Sp)**: the smallest number of samples needed to create a split;
  - **Minimum samples required for a leaf node (Lf)**: the minimum number of samples required to form a leaf node;
  - **Maximum number of features considered for each tree subset (N_f_)**: the number of features randomly selected for splitting at each node;
  - **Splitting criterion (crit)**: the metric used to evaluate the quality of a split (e.g., Gini impurity or entropy).
    Table S1 provides information on the range of possible parameterized values for each hyperparameter subjected to tuning. This systematic hyperparameter optimization enabled the random forest model to achieve high performance and robustness while maintaining generalizability, even with the limited dataset size.
4. **GA Optimization:** A GA was used to optimize the hyperparameters of RFE and the random forest. The algorithm is based on the evolution of a population (in this case, a set of hyperparameters), mimicking genetic processes such as crossover and mutation to maximize the population’s fitness to the given conditions with each successive generation. The schematic of the applied GA is presented in Figure 1. The process began with an initial population of 100 randomly generated hyperparameter sets. For each set, RFE and a random forest were executed and the MCC was calculated to evaluate model performance. The fitness of each model was determined using the following formula:
  Fitness = MCC^15^ · N_RFE_^2^ (1)
  The fitness function was designed to strongly prioritize hyperparameter sets achieving an MCC of 1.0. An exponent of 15 was applied to the MCC, resulting in models with an MCC of 1.0 having weights approximately five times greater than those with an MCC of 0.9. Additionally, the function promoted models retaining more metabolite bins after RFE by incorporating a quadratic exponent for N_RFE_.
  The fitness values of the population were transformed into weights, representing the probability of selecting each member as a parent for the next generation. Based on these weights, N_par_ = 2 parents was randomly selected with the replacement for crossover. During the crossover process, N_cross_ = 2 hyperparameters was randomly exchanged between paired members. This procedure was repeated until N_par_ − 2⋅N_elite_ = 96 new members was generated. To ensure performance preservation, N_elite_ = 2 top-performing members was directly carried over to the next generation.
  Next, point mutations were applied to one hyperparameter (N_mut_ = 1) of the remaining population members, with a mutation probability (P_mut_) of 90%. Finally, a duplicate copy of the N_elite_ members was carried over to the next generation without modification, ensuring that the new generation once again consisted of N_pop_ = 100 members.
  The procedure was executed N_gen_ = 35 times, continuing until the evolutionary progress appeared to converge, as illustrated in Figure S1. This process facilitated the exploration of approximately 2500 hyperparameter combinations, sampled from a total search space of over 6.7 million predefined possibilities. The iterative approach sought to identify a suboptimal hyperparameter set by leveraging successive cycles of fitness evaluation, crossover, mutation, and elite preservation.

### 2.4. Feature Importance Analysis

To further analyze the contributions of each feature, several feature importance metrics were applied post-modelling. These included the following:

**Centroid Distance Calculation:** The Euclidean distance between OA- and RA-group centroids was calculated for each metabolite bin, serving as a metric for group separability.

**Fisher Discriminant Ratio (FDR):** The FDR was computed to evaluate the ability of metabolite bins to discriminate between two classes. It is defined as the ratio of the between-class variance (i.e., the squared distance between class centroids) to the within-class variance, providing a quantitative measure of the feature’s discriminatory power [22].

**SHAP (Shapley Additive Explanations) Analysis:** SHAP values were computed for the most effective model to interpret the contributions of individual metabolite bins to its predictions. Specifically, SHAP values were calculated for each metabolite bin retained after RFE (i.e., metabolite bins for which the MCC after the decision stump did not reach 1.0) to identify those metabolite bins that most significantly influenced the classification of patients into one of the disease categories. This analysis not only highlighted key contributors to the classification but also enabled a qualitative examination of potential correlations between metabolites, offering deeper insights into their inter-relationships.

## 3. Results

### 3.1. Data Preprocessing and Exploratory Data Analysis

Our primary goal was to determine which scaling method would yield the best separation of patients into OA and RA classes. To assess data separation, we employed the silhouette score. Additionally, for better interpretability, we analyzed the eigenvalues of the two most critical principal components in the PCA.

We evaluated five different normalization methods, with min–max scaling and median + Pareto scaling emerging as the top-performing approaches. As shown in Figure S2, the results of the PCA revealed that min–max scaling explained 37.9% and 29.0% of the variance in the first two principal components (PC1 and PC2, respectively). In comparison, median + Pareto scaling captured a higher proportion of variance, explaining 62.0% of the variance in PC1 and 15.2% in PC2. However, when evaluating clustering performance using the silhouette score, min–max scaling outperformed median + Pareto scaling, achieving a higher silhouette score of 0.2729 compared with 0.2193 respectively. This indicated that min–max scaling provided better clustering performance and more distinct separation between the two study groups.

Given the importance of group separability in this study, the silhouette score was prioritized as the key criterion for selecting the normalization method. Based on this metric, min–max scaling was identified as the optimal approach for downstream analyses.

**Figure 2:**
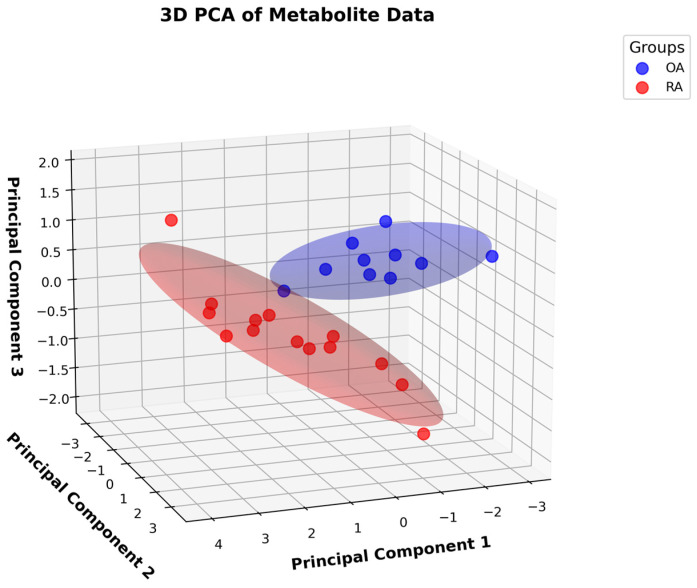
A 3D principal component analysis (PCA) of metabolite data differentiating osteoarthritis (OA) and rheumatoid arthritis (RA) samples.

Although a 2D PCA provides a simplified visualization, it may not sufficiently capture a dataset’s complexity and separate the groups effectively. To address this, we extended the analysis to a 3D PCA to determine whether adding a third dimension would improve the resolution of the separation and better highlight the underlying data structure. As shown in Figure 2, the 3D PCA revealed a clearer separation between the OA and RA samples, providing additional insight into the dataset’s structure for further examination. The first three principal components explained 75% of the total variance, demonstrating that they captured a substantial portion of the dataset’s structure.

Despite the apparent separation in 3D, the original metabolite bin set was retained for subsequent analyses. Although the PCA provided valuable insights as a visualization and exploratory tool, its limitations became apparent in the 3D projection. The separation, whilst promising, was somewhat confused by overlapping points, causing the clusters to touch. This overlap underscored the need for more robust analytical methods to better disentangle the underlying relationships. As a result, we explored additional approaches, including a PLS-DA, to achieve clearer separation and leverage the full predictive potential of the data.

Subsequently, a PLS-DA was applied to verify whether strong clustering existed between OA and RA patients. As shown in Figure S3, OA points were distinctly concentrated in one region, while RA points, although more dispersed, still showed clear clustering for both classes. This analysis further supported the choice of min–max scaling as the optimal normalization method.

We then sought to identify which metabolite bins most significantly influenced the class separation. The PLS-DA highlighted several metabolite bins with notable class-specific variations, as shown in Table 1. Specifically, high normalized levels of pyruvic acid, L-glutamine, taurine, adenosine, L-lysine, D-glucose, citric acid, L-tyrosine, and 3-hydroxyisovaleric acid were associated with the classification of patients having OA. In contrast, elevated concentrations of L-proline, L-threonine, sarcosine, 3-hydroxybutyric acid, glycine, isopropyl alcohol, L-isoleucine, acetic acid, phosphorylcholine, and L-methionine were indicative of the RA classification. The number in square brackets in Table 1 represents the mean of the range of the bin.

### 3.2. Machine-Learning-Based Classification and Feature Selection

The growing interest in the application of machine learning (ML) in research, particularly in disease diagnostics, motivated us to explore its broader utility [23,24,25,26]. Our goal was to develop a simple-to-implement algorithm to identify metabolites most strongly associated with the presence of specific diseases in patients. Additionally, we aimed to compare these findings with conventional techniques such as the previously applied PLS-DA. Using a supervised machine learning approach, we developed a workflow to systematically select and analyze relevant metabolites (Figure 1).

In the initial step, metabolite bins with a clear decision boundary—defined as having an MCC (Matthews correlation coefficient) value of 1.0, a robust metric that accounts for both balanced accuracy and error distribution—were selected for further analyses. This process identified bins from four metabolites meeting the criterion, including L-glutamine, L-proline, pyruvic acid, and acetic acid.

**Figure 3:**
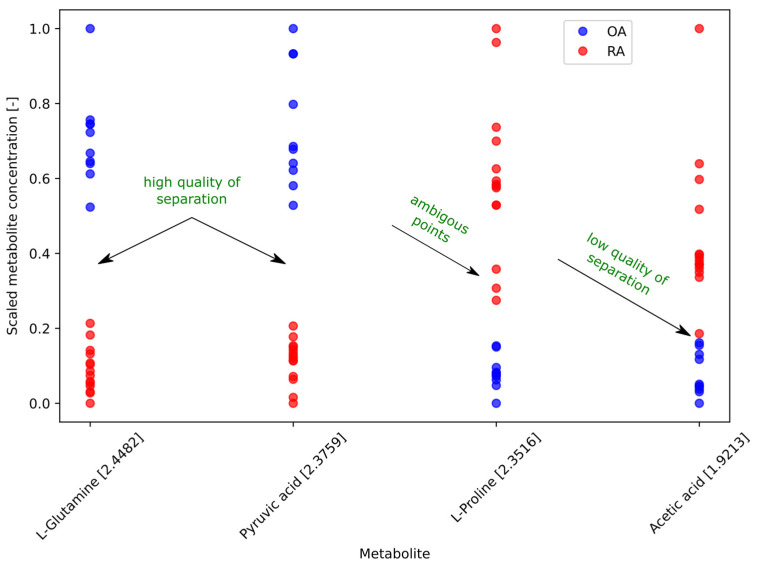
The 1D scatter plots of the most discriminative metabolite bins for differentiating osteoarthritis (OA) and rheumatoid arthritis (RA) samples. Each plot displays the distribution of metabolite levels, normalized using min–max scaling.

To evaluate the quality of data separation into two clusters using a simple decision stump, we analyzed the four identified metabolite bins. As shown in Figure 3, L-glutamine and pyruvic acid exhibited two distinctly separated clusters. These results suggest that high concentrations of these metabolites were indicative of OA, while low concentrations pointed toward RA.

For L-proline, a clear decision boundary was observed, with lower concentrations aligning with OA and higher concentrations with RA. However, it is important to note that the decision stump algorithm is a supervised method. Without predefined labels, the classification of three points with normalized concentrations between 0.2 and 0.4 would remain ambiguous (see Figure 3). In the case of acetic acid, one point classified as RA was positioned closer to the OA cluster, which, like L-proline, could present challenges for unsupervised classification.

To numerically assess the separation quality between clusters, we calculated the Euclidean distance between cluster centroids. As shown in Table 2, the distances for L-glutamine and pyruvic acid exceeded 0.6, while for L-proline, the distance was slightly above 0.5; for acetic acid, it was around 0.4. However, given the significant differences in data distributions across classes (illustrated in Figure 3), the Euclidean distance alone may not have been a sufficiently robust metric.

To complement this analysis, we examined the standard deviations of each metabolite, as presented in Table 2. Additionally, we applied the three-sigma rule, following the approach in [27], to assess the quality of cluster separation. For L-glutamine and pyruvic acid, none of the three-fold standard deviations exceeded the centroid distance, indicating strong separation. In contrast, for L-proline and acetic acid, at least one of the three-fold standard deviations surpassed the centroid distance, suggesting weaker separation.

To further refine our assessment, we computed the Fisher discriminant ratio (FDR), which considers both inter-group variance and intra-group variability. This metric provides a more comprehensive basis for determining whether the observed distances correspond with well-separated clusters, offering a stronger justification for assessing separation quality [27].

Table 2 highlights the FDR values. L-glutamine and pyruvic acid showed high scores of 19.24 and 12.77, respectively, reflecting strong class separation. In contrast, L-proline and acetic acid had significantly lower FDR values of 5.66 and 3.36, respectively, indicating weaker separation. These results suggest that while L-glutamine and pyruvic acid provided reliable differentiation between classes, the separation for L-proline and acetic acid was relatively limited.

Thus, our approach not only identified metabolite bins with clear decision boundaries but also assessed their significance using metrics that considered both cluster distances and intra-cluster variance.

**Figure 4:**
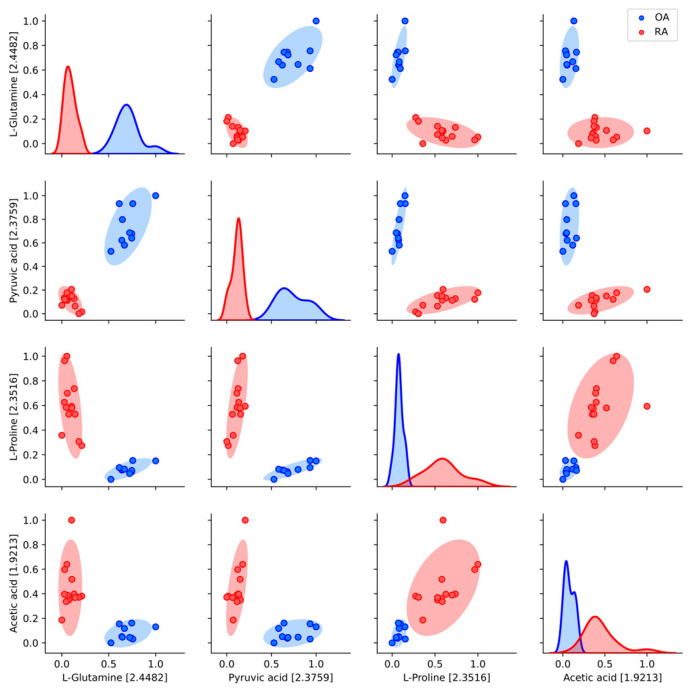
Pair plots of metabolite distributions and relationships by cluster. Diagonal plots show the distribution of each metabolite bin within the respective clusters (OA and RA), while the off-diagonal plots display pairwise relationships and distributions of metabolites, categorized by their assigned clusters. Each scatter plot highlights the separability and potential correlations between metabolites. Clustering was performed based on scaled data.

We also aimed to investigate whether using multiple metabolite bins in the analysis could improve the quality of data separation, based on the four previously identified metabolites. The qualitative assessment, illustrated in Figure 4, demonstrated that combining two metabolites significantly enhanced cluster separability. This effect was particularly noticeable when examining combinations such as L-proline and acetic acid, where the synergy between the two features enabled better differentiation between disease classes compared with individual metabolites. These results emphasize the usefulness of multi-feature analyses in uncovering patterns within metabolite data. Building on these findings, we proceeded to apply the random forest model to analyze metabolite bins with Matthews correlation coefficient (MCC) values below 1.0.

Before the data were classified using the random forest, we applied RFE to select the most relevant bins. As the PCA indicated high linear separability between classes, a support vector machine (SVM) with a linear kernel was iteratively used to train the model, progressively eliminating the least significant bins. This process culminated in identifying N_RFE_ = 50 significant metabolite bins. This step helped to eliminate less discriminative features that could introduce noise and undermine the robustness of the classification.

We trained a random forest model to achieve an MCC of 1.0 using leave-one-out cross-validation (LOOCV). To optimize the model, we employed a genetic algorithm, which identified the best-performing configuration (highest fitness value). The resulting model hyperparameters were as follows:

- N_est_ = 100 trees;
- Maximum tree depth D = 6;
- Minimum samples per split Sp = 10;
- Minimum samples per leaf Lf = 6;
- Maximum features per tree N_f_ = ⌊log_2_(N_RFE_)⌋ = 5,
- A ‘log_loss’ split criterion.

We observed that the high values of Sp and Lf relative to the small sample size of 24 indicated that these parameters played a significantly larger role in hyperparameter tuning compared with D. This also suggested a preference for trees with limited branching, which helped to prevent overfitting.

To evaluate the impact of RFE, we first assessed model performance with feature selection where all metrics—MCC, accuracy, precision, recall, F1-score, and ROC-AUC—reached 1.0. In contrast, when RFE was not applied, performance declined, yielding an MCC of 0.74, an accuracy of 0.88, a precision of 0.87, a recall of 0.93, an F1-score of 0.90, and a ROC-AUC of 0.86. This evaluation underscored the extent to which the inclusion of irrelevant metabolite bins impaired classification performance. Even with an increased number of N_est_ (Figure S4), it became evident that achieving an MCC of at least 0.85 for this dataset was unattainable. This limitation arose from the fact that several metabolite bins used in the decision stump analysis exhibited MCC values close to or equal to 0 (Table S2), indicating similarity with random guessing. Their inclusion in the model substantially reduced its effectiveness.

Therefore, we concluded that using RFE in advance was an excellent way to achieve more precise results by eliminating features that introduced noise rather than meaningful separation.

**Figure 5:**
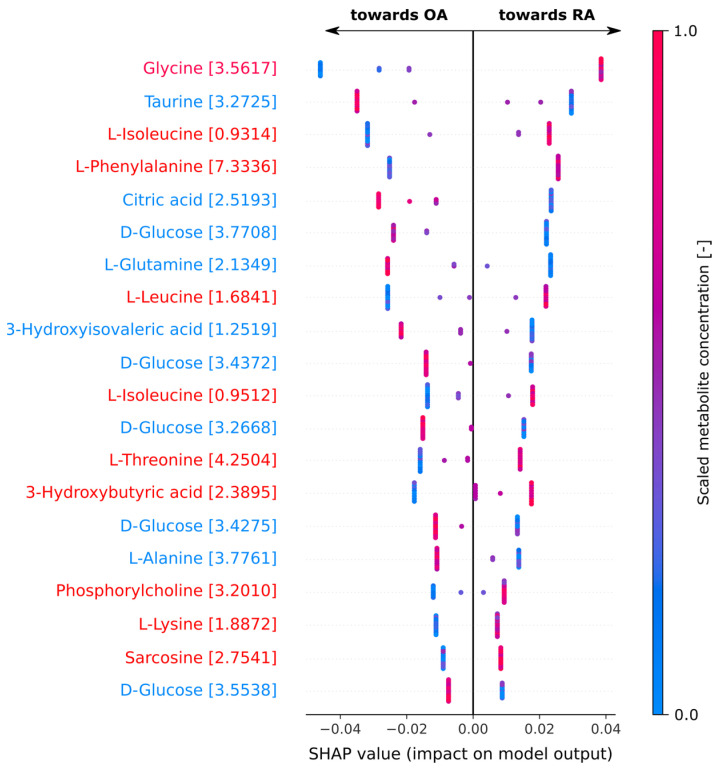
SHAP analysis of the top 20 most key metabolite bins for disease classification in a random forest. Positive SHAP values indicate a higher likelihood of the patient being classified as having RA, while negative values suggest a higher likelihood of classification as OA. Metabolites with higher concentrations that promote RA are marked in red, while those promoting OA are marked in blue.

Like the PLS-DA, we aimed to identify the metabolites that most strongly influenced the classification of patients as OA or RA using our decision-tree-based classifier. For the best-performing model, we conducted a SHAP (Shapley additive explanations) analysis to assess the contribution of individual bins to the classification. The SHAP values for the most critical metabolite bins are visualized in Figure 5.

The analysis revealed that elevated levels of glycine, L-isoleucine, L-phenylalanine, and L-leucine were strongly associated with an RA diagnosis. This suggests that nonpolar or aromatic amino acids are particularly linked to RA. However, it is also important to note that polar compounds such as L-threonine, 3-hydroxybutyric acid, and phosphorylcholine are also associated with the disease.

Conversely, higher levels of taurine, citric acid, D-glucose, and L-glutamine, which are predominantly polar compounds, were more indicative of OA. It is also noteworthy, as shown in Figure 5, that bins corresponding with D-glucose appeared as often as five times in the analysis of the 20 most important bins identified using SHAP. This not only highlighted the significant role of D-glucose in OA diagnoses but also underscored its utility as a valuable marker for disease detection.

## 4. Discussion

### 4.1. Diagnostic Implications of Metabolite Selection

Metabolomics is a rapidly evolving field that offers valuable insights into disease mechanisms. By identifying metabolic biomarkers, this approach enables early and accurate diagnosis, supports timely interventions, and provides tools to monitor disease progression and assess treatment outcomes [23,24]. Osteoarthritis (OA), increasingly recognized for its metabolic underpinnings, currently lacks pharmacologic therapies that can halt or reverse its progression [17,18,19,20]. Similarly, rheumatoid arthritis (RA) is characterized by distinct metabolic alterations linked to its inflammatory nature [13,17]. Identifying specific metabolic biomarkers in RA can facilitate early diagnosis, predict disease severity, and tailor treatments more effectively.

The early detection of both OA and RA is crucial to mitigating joint damage, addressing modifiable risk factors, and optimizing long-term outcomes. By leveraging metabolomics to uncover these biomarkers, we can bridge the gap between fundamental biochemical insights and their application in personalized medicine. This approach underscores the transformative potential of metabolomics in advancing both clinical practice and research innovation.

**Figure 6:**
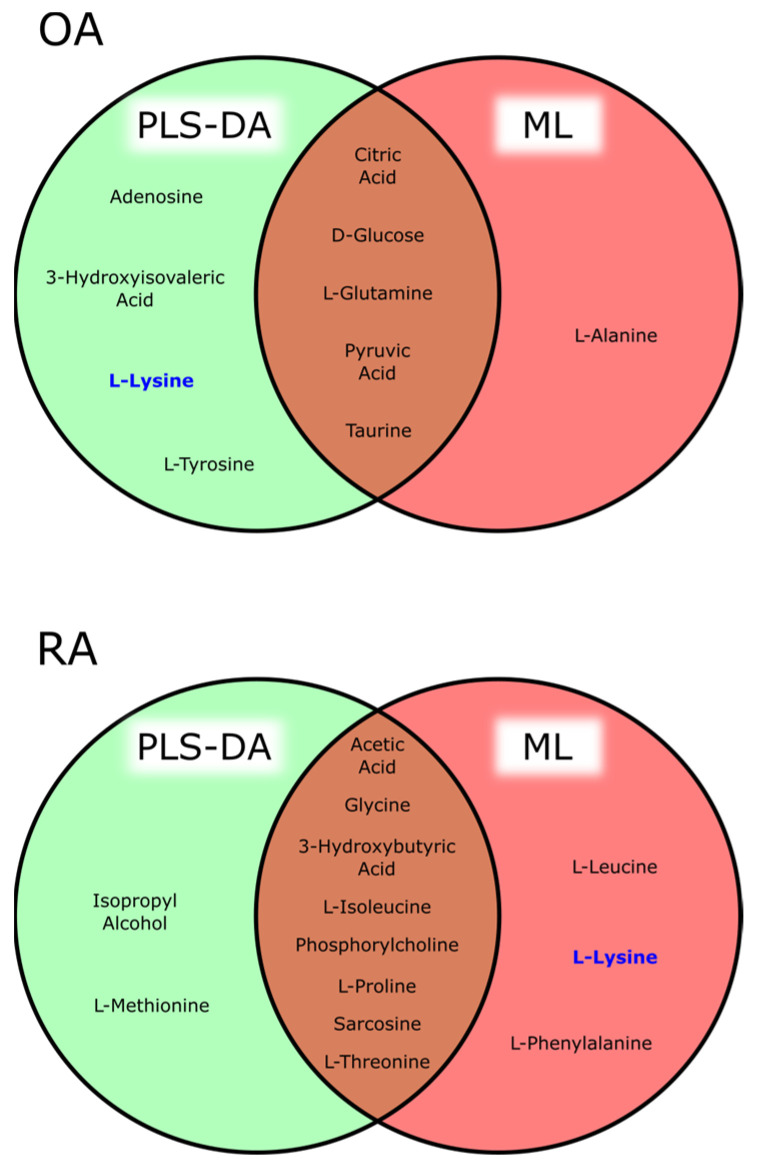
Venn diagrams illustrating key metabolites identified for the diagnosis of OA (**top**) and RA (**bottom**) using both PLS-DA and machine-learning-based approaches. The blue color indicates a metabolite ambiguously associated with both diseases.

The Venn diagrams presented in Figure 6 illustrate the key metabolites identified for diagnosing OA and RA using two distinct analytical approaches, a PLS-DA and ML. This comparative analysis highlights both the overlap and divergence in metabolite selection as well as metabolites that exhibit ambiguous associations with both diseases.

For OA, several metabolites, including citric acid, D-glucose, L-glutamine, pyruvic acid, and taurine, were consistently identified by both PLS-DA and ML-based approaches. This overlap underscored their robustness as diagnostic markers, demonstrating reliability across distinct analytical frameworks. In contrast, unique findings emerged from each method. The PLS-DA exclusively identified metabolites such as adenosine, 3-hydroxyisovaleric acid, and L-tyrosine, highlighting their potential relevance to OA pathophysiology through the discriminatory capabilities of this multivariate approach. Meanwhile, ML-based methods uniquely pinpointed L-alanine, suggesting its potential as a distinct biomarker detected through advanced algorithmic selection. Interestingly, L-lysine emerged as an ambiguous metabolite associated with both OA and RA, suggesting a shared metabolic pathway or common inflammatory process that could underlie both diseases [25].

The recurrent significance of D-glucose and citric acid in OA pointed to disrupted energy pathways, which may be associated with cartilage degeneration and altered cellular energy demands. These findings aligned with the well-established role of energy metabolism dysregulation in OA progression [26]. It is also worth noting from the SHAP analysis that the effective diagnosis of OA was influenced by the analysis of five distinct NMR bins, further emphasizing that D-glucose was a crucial and easily identifiable biomarker for disease identification. Additionally, taurine’s significant contribution toward OA classification, as indicated by its large negative SHAP values, underscored its potential as a biomarker for identifying or monitoring OA. Taurine, a well-known regulator of osmoregulation and antioxidant responses, may play a crucial role in protecting cartilage from oxidative stress and inflammation, further emphasizing its importance in the pathophysiology of OA [28].

For RA, several metabolites were consistently identified by both the PLS-DA and ML-based methods, including acetic acid, glycine, 3-hydroxybutyric acid, L-isoleucine, phosphocholine, and L-threonine. This overlap underscored their reliability as biomarkers for RA, demonstrating their robustness across distinct analytical frameworks. Elevated levels of amino acids such as glycine and L-phenylalanine point to metabolic shifts related to protein catabolism or inflammatory responses, hallmark features of systemic autoimmune conditions [29]. Additionally, metabolites like phosphorylcholine and sarcosine may reflect increased lipid turnover and oxidative stress, which are commonly observed in RA due to chronic systemic inflammation. Distinct findings further emphasized the strengths of each method. The PLS-DA uniquely highlighted isopropyl alcohol and L-methionine, underscoring their potential relevance in the autoimmune mechanisms of RA. In contrast, the ML approach identified L-leucine and L-phenylalanine as distinct biomarkers, highlighting the ability of advanced algorithmic methods to pinpoint unique metabolic features. Together, these findings support the hypothesis that RA pathogenesis is driven by extensive alterations in lipid metabolism and oxidative damage, offering valuable insights into its metabolic underpinnings [30].

The identification of L-lysine as a shared metabolite between OA and RA is particularly noteworthy. This overlap could reflect metabolic disturbances common to both diseases, such as those associated with inflammation, tissue remodeling, or energy metabolism [31]. The presence of L-lysine in both disease states complicates its utility as a disease-specific biomarker and warrants further investigation to discern its precise role and context-dependent relevance.

The integration of machine learning and genetic algorithm techniques optimized disease classification performance while ensuring model simplicity and accuracy, providing a deeper understanding of the critical metabolites involved [32,33]. The decision stump model, combined with centroid distance and Fisher discriminant ratio metrics, allowed us to identify the most critical metabolites for classification. Additionally, these methods provided valuable insights into the reliability of class separation, facilitating a critical assessment of how individual metabolites influenced patient classification. The combination of RFE and SVM effectively retained the most relevant features for classification. This approach significantly reduced the complexity of the random forest model, requiring fewer estimators and simplifying the decision trees while maintaining 100% classification accuracy.

### 4.2. Limitations and Considerations

Although the study successfully demonstrated the efficacy of selected metabolites in OA and RA classification, the analysis relied on data obtained from a single source (MetaboLights dataset MTBLS564). The model’s generalizability could benefit from further validation across diverse datasets and patient cohorts to ensure robustness. Additionally, although NMR spectroscopy facilitated metabolite identification, the method’s sensitivity could vary across clinical laboratories, suggesting that further cross-platform validation (e.g., with mass spectrometry techniques) would enhance the reproducibility of the findings.

Another limitation of this study was the relatively small sample size, which may have affected the stability and generalizability of the classification model. Although we did not apply any data augmentation techniques in our analysis, the existing literature provides potential solutions for small datasets. For instance, the study by Ivan Izonin et al. proposes an approach that generates synthetic data points closely resembling the original distribution [34]. Such methods could be explored in future research to enhance the robustness of small-scale metabolomic studies.

### 4.3. Comparison with Existing Literature

This work extended previous research by implementing an advanced, feature-optimized random forest model that surpassed traditional PCA and PLS-DA approaches in diagnostic power. Previous studies using NMR and machine learning have identified metabolites relevant to OA and RA, yet few have reduced the diagnostic process to as few as five key markers. Compared with the broader metabolite panels reported in studies like those by Anderson et al. and Bocsa et al., the targeted selection in this study provided a streamlined workflow potentially adaptable to clinical practice [17,18].

The results of the current study show a significant level of concordance with the findings reported in the study by Anderson et al., particularly regarding the metabolites identified as differing between OA and RA [17]. Both studies highlight key metabolic distinctions that provide valuable insights into the pathophysiology of these diseases.

Several metabolites identified as being significantly altered in the study by Anderson et al. were also consistent with our findings. For instance, in OA, metabolites such as citric acid, D-glucose, L-glutamine, pyruvic acid, and taurine were consistently identified as elevated. These findings aligned with the report by Anderson et al. of these metabolites being significantly higher in OA, reinforcing their relevance in energy metabolism and cartilage homeostasis. In RA, metabolites such as acetic acid, glycine, L-isoleucine, and L-threonine were similarly identified as elevated in both studies. This consistency underscores their robustness as biomarkers of systemic inflammation and protein catabolism.

However, some divergences were observed. In the study by Anderson et al., proline was significantly higher in OA. In contrast, our study identified L-proline as being associated with RA, as indicated by its positive PLS coefficient in our analysis. Similarly, 3-hydroxybutyric acid also showed differing results between the studies. Anderson et al. reported it as being significantly higher in OA, whereas our study identified it as being elevated in RA. This discrepancy could stem from differences in analytical approaches, highlighting the complexity of metabolite behavior across different study designs.

Notably, our study also uniquely identified L-lysine as a shared metabolite associated with both OA and RA. This finding, not reported in the study by Anderson et al., suggests a potential overlap in inflammatory or tissue remodeling pathways common to both diseases. However, its presence in both conditions complicates its role as a disease-specific biomarker and warrants further investigation. Finally, our study expanded on Anderson et al.’s findings by employing machine learning (ML)-based approaches, which identified unique metabolites not highlighted in the earlier study. For example, L-phenylalanine and phosphorylcholine were uniquely linked to RA, demonstrating the added discriminatory power of advanced algorithms.

In summary, although our findings aligned with those of the dataset owners, they also extended the understanding of OA and RA metabolomics by identifying additional metabolites and providing insights into shared and distinct metabolic pathways. Divergences, such as proline and 3-hydroxybutyric acid, underscore the importance of further research to better understand these metabolic variations and their implications.

## 5. Conclusions

This study integrated findings from multiple analytical approaches and compared them with existing literature, offering a more comprehensive understanding of the metabolic landscape of OA and RA. It validated well-established pathways, introduced novel biomarkers, and highlighted the crucial role of advanced methodologies in enhancing our understanding of arthritis pathogenesis. Moving forward, research should focus on the translational potential of these biomarkers for early diagnosis, disease monitoring, and targeted therapies. Additionally, multi-cohort validation is necessary to confirm the generalizability of these findings, potentially broadening the model’s applicability across various stages of OA and RA.

## Figures and Tables

**Table 1:** The most discriminative metabolites contributing to class separation based on PLS coefficients.

	Metabolite Bins Associated with the Classification of Patients Having OA		Metabolite Bins Associated with the Classification of Patients Having RA	
Rank	Name	PLS Coefficient	Name	PLS Coefficient
1	Pyruvic Acid [2.3759]	−0.028	L-Proline [2.3516]	0.024
2	L-Glutamine [2.4482]	−0.026	L-Threonine [4.2504]	0.022
3	L-Glutamine [2.1349]	−0.024	Sarcosine [2.7541]	0.020
4	Taurine [3.2725]	−0.019	3-Hydroxybutyric Acid [2.3895]	0.019
5	Adenosine [8.2113]	−0.019	Glycine [3.5617]	0.018
6	L-Lysine [3.0480]	−0.017	Isopropyl Alcohol [4.0255]	0.017
7	D-Glucose [3.7708]	−0.017	L-Isoleucine [0.9314]	0.017
8	Citric Acid [2.5193]	−0.016	Acetic Acid [1.9213]	0.016
9	L-Tyrosine [6.9040]	−0.016	Phosphorylcholine [3.2010]	0.016
10	3-Hydroxyisovaleric Acid [1.2519]	−0.016	L-Methionine [2.6397]	0.016

**Table 2:** Key metrics for four distinctive metabolite bins in OA and RA.

	L-Glutamine [2.4482]	Pyruvic Acid [2.3759]	L-Proline [2.3516]	Acetic Acid [1.9213]
Distance between centroids	0.62	0.63	0.52	0.37
Standard deviations for each cluster	0.13/0.06	0.17/0.06	0.05/0.21	0.06/0.19
Fisher’s discriminant ratio (FDR)	19.24	12.77	5.66	3.36

## Data Availability

The original contributions presented in the study are included in the article/supplementary material, further inquiries can be directed to the corresponding author/s. Metabolomics data is publicly available in the EMBL-EBI MetaboLights database with the identifier MTBLS564. The complete data set can be accessed at https://www.ebi.ac.uk/metabolights/MTBLS564.

## Supplementary Materials

The following supporting information can be downloaded at: https://www.mdpi.com/article/10.3390/metabo15020112/s1, Figure S1: Progression of model hyperparameters through generations, with average MCC and fitness values shown for each generation; Table S1: Hyperparameters optimized using a genetic algorithm and an exploration of the corresponding value ranges; Figure S2: The 2D PCA plots for different scaling methods, showing cluster distributions. The upper right corner of each plot displays the cumulative variance explained by the first two principal components and the silhouette score. Bold values indicate the highest metric for each method; Table S2: Metabolites with highest and lowest MCC values in decision stump analysis; Figure S3: PLS-DA applied to min–max scaled data; Figure S4: MCC values as a function of the number of estimators (Nest) for the random forest model without applying recursive feature elimination (RFE) to exclude the least-significant metabolites. The upper right corner shows the hyperparameters of the model that achieved MCC = 1.0 when RFE was applied.

## Abbreviations

The following abbreviations are used in this manuscript:

OA	Osteoarthritis
RA	Rheumatoid arthritis
ML	Machine learning
SF	Synovial fluid
NMR	Nuclear magnetic resonance
PCA	Principal component analysis
PLS-DA	Partial least squares discriminant analysis
GA	Genetic algorithm
SHAP	Shapley additive explanations
RF	Rheumatoid factor
ACPA	Anti-citrullinated protein antibodies
LC-MS	Liquid chromatography coupled with mass spectrometry
RF	Random forest
BFS	Boruta feature selection
ST	Synovial tissue
RFE	Recursive feature elimination
SVM	Support vector machine
LOOCV	Leave-one-out cross-validation
MCC	Matthews correlation coefficient
FDR	Fisher discriminant ratio
