# Supplementary material for: Advanced Machine Learning for Comparative Synovial Fluid Analysis in Osteoarthritis and Rheumatoid Arthritis
Source: Metabolites. 2025 Feb 10;15(2):112. doi: 10.3390/metabo15020112 (PMC11857505; doi:10.3390/metabo15020112)
Supplement: Supplementary file 1 [file metabolites-15-00112-s001.zip › SI_Advanced Machine Learning for Comparative Synovial Fluid Analysis in Osteoarthritis and Rheumatoid Arthritis.pdf]

Table S1. Hyperparameters optimized through a genetic algorithm and the corresponding value ranges explored.

lp.	Hyperparameter	Algorithm Component	Search Range	Range Size
1	N_{RFE}	RFE	15-50 (step: 1)	36
2	N_{est}	Random Forest	50-1000 (step: 25)	39
3	D	Random Forest	None, 1-10 (step: 1)	11
4	Sp	Random Forest	2-10 (step: 1)	9
5	Lf	Random Forest	1-8 (step: 1)	8
6	N_f	Random Forest	[‘sqrt’, ‘log2’]	2
7	crit	Random Forest	[‘gini’, ‘entropy’, ‘log_loss’]	3

Table S2. Metabolites with Highest and Lowest MCC Values in Decision Stump Analysis

	Most disciriminative metabolite		Least disciriminative metabolite	
**Rank**	**Name**	**MCC**	**Name**	**MCC**
1	Acetic Acid [1.9213]	1.00	Glycerol [3.5576]	0.00
2	L-Proline [2.3516]	1.00	Myoinositol [3.2915]	0.00
3	Pyruvic Acid [2.3759]	1.00	Citric Acid [2.6702]	0.00
4	L-Glutamine [2.4482]	1.00	L-Isoleucine [1.9642]	0.00
5	L-Glutamine [2.1349]	0.92	α-Hydroxyisobutyric Acid [1.5808]	0.00
6	Taurine [3.2725]	0.92	Adenosine [8.3370]	0.25
7	D-Glucose [3.7708]	0.85	L-Histidine [7.8746]	0.25
8	L-Isoleucine [0.9314]	0.85	D-Glucose [3.7570]	0.25
9	Citric Acid [2.5193]	0.85	Glycerol [3.6501]	0.30
10	Sarcosine [2.7541]	0.85	Choline [3.1920]	0.31

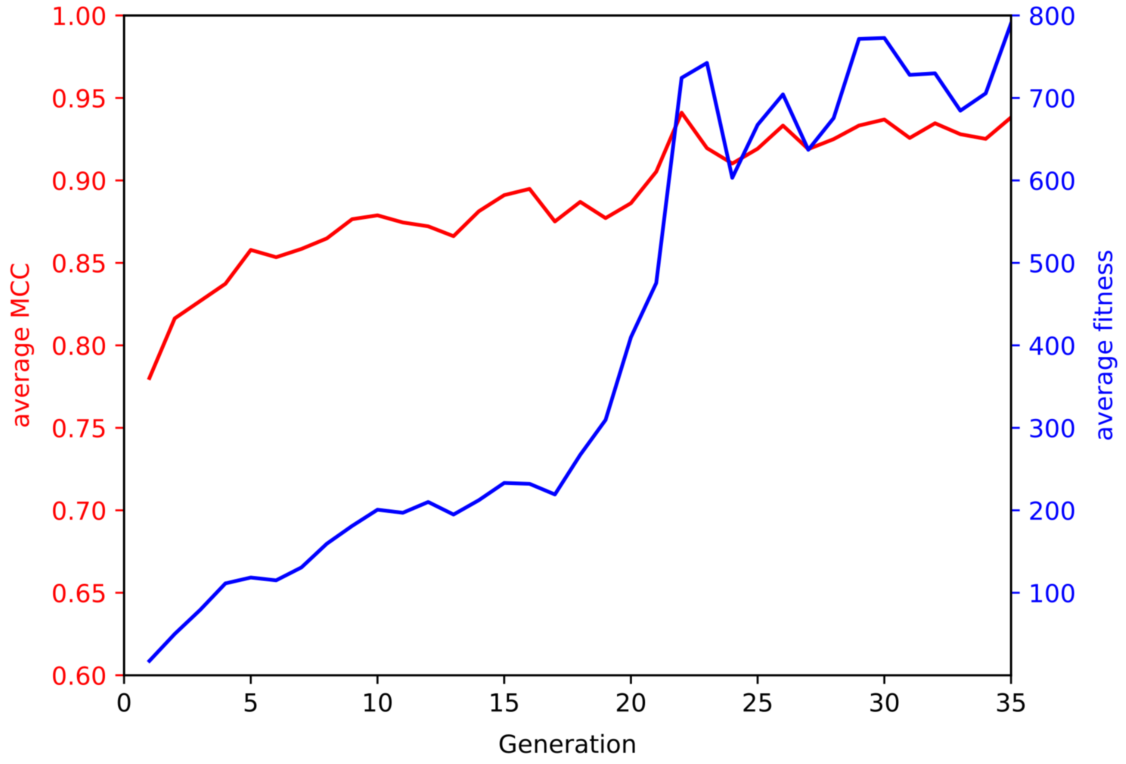

Figure S1. Progression of model hyperparameters through generations, with average MCC and fitness values shown for each generation.

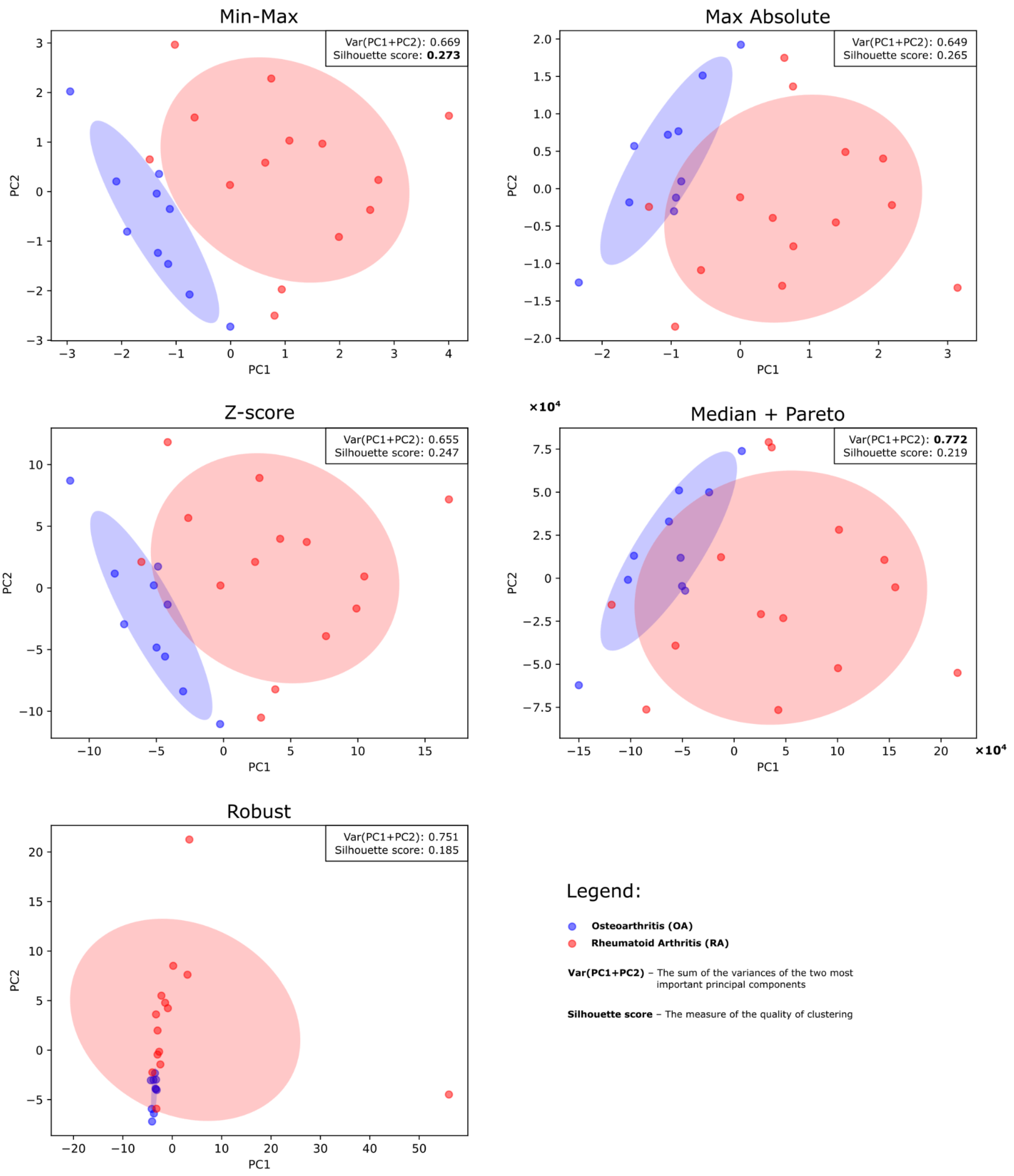

Figure S2. 2D PCA plots for different scaling methods, showing cluster distributions. The upper right corner of each plot displays the cumulative variance explained by the first two Principal Components and the Silhouette Score. Bolded values indicate the highest metric for each method.

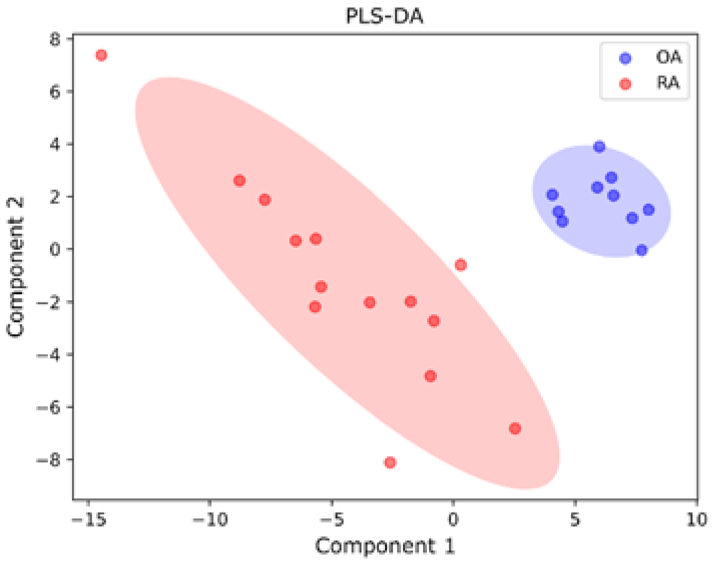

Figure S3. PLS-DA applied to the Min-Max scaled data.

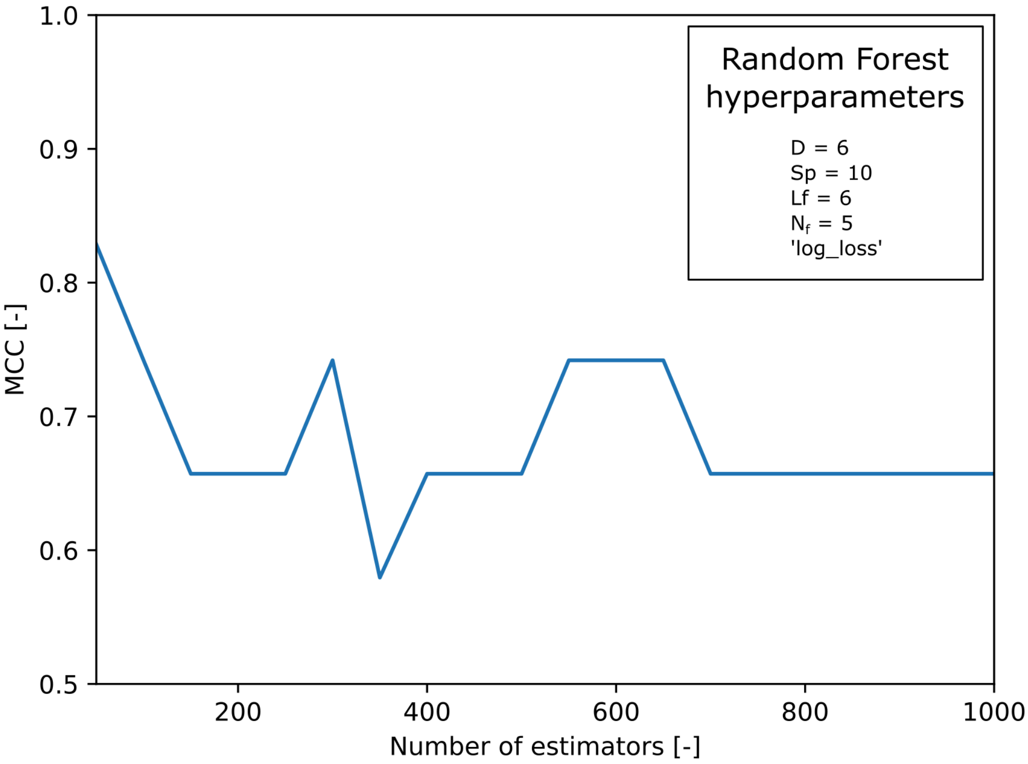

Figure S4. MCC values as a function of the number of estimators (Nest) for the Random Forest model without applying Recursive Feature Elimination (RFE) to exclude the least significant metabolites. The upper right corner shows the hyperparameters of the model that achieved MCC = 1.0 when RFE was applied.
